# Characteristics of CD8+ T cell subsets in Chinese patients with chronic HIV infection during initial ART

**DOI:** 10.1186/1742-6405-8-15

**Published:** 2011-03-25

**Authors:** Yanmei Jiao, Wei Hua, Tong Zhang, Yonghong Zhang, Yunxia Ji, Hongwei Zhang, Hao Wu

**Affiliations:** 1 Center for Infectious Diseases, Beijing You'an Hospital, Capital Medical University, Beijng 100069, China

## Abstract

**Background:**

CD8+ T cells may play an important role in protecting against HIV. However, the changes of CD8+ T cell subsets during early period of ART have not been fully studied.

**Methods:**

Twenty-one asymptomatic treatment-naive HIV-infected patients with CD4 T+ cells less than 350 cells/μl were enrolled in the study. Naïve, central memory(CM), effective memory(EM) and terminally differentiated effector (EMRA) CD8+ cell subsets and their activation and proliferation subsets were evaluated in blood samples collected at base line, and week 2, 4, 8 and 12 of ART.

**Results:**

The total CD8+ T cells declined and the Naïve and CM subsets had a tendency of increase. Activation levels of all CD8+ T cell subsets except EMRA subset decreased after ART. However, proliferation levels of total CD8+ T cells, EMRA, EM and CM subsets increased at the first 4 weeks of ART, then decreased. Proliferation level of the naïve cells decreased after ART.

**Conclusion:**

The changes of CD8+ T cell subsets during initial ART are complex. Our results display a complete phenotypical picture of CD8+ cell subsets during initial ART and provide insights for understanding of immune status during ART.

## Background

CD8+ T cells play an important role in protection against intracellular pathogens. Eliminating CD8+ T lymphocytes from monkeys during chronic SIV infection resulted in a rapid and marked increase in viremia, which was again suppressed coincident with the reappearance of SIV-specific CD8+ T cells. Antiviral CD8+ T cells controlled the acute viremic phase of the infection, resulting in the establishment of the viral set point [1-3].

Many studies [4-6] evaluated the changes of CD4+ cell subsets during antiretroviral treatment (ART). However, the changes of CD8+ T cell subsets in early period of ART have not been fully studied yet.

Here, in our study, we investigated the characteristics of naive (CD45RA +CCR7+), central memory (CM) (CD45RA - CCR7+), effector memory (EM) (CD45RA- CCR7-), and terminally differentiated effector (EMRA)(CD45RA+ CCR7-) cell subsets[7,8], as well as activation and proliferation levels of each subset, during initial ART in Chinese patients. Our results demonstrated that most of the CD8+ cell subsets decrease during initial ART, while Naïve and CM subsets have a tendency of increase, which may reflect the immune reconstitution of CD8+ T cells.

## Results

### Baseline demographic and clinical characteristics of the subjects

A total of 21 HIV/AIDS patients were enrolled from Beijing You'an Hospital, Capital Medical University. At baseline, the average age of subjects was 36.8 ±12.1 years (range, 23-64 years). The median plasma viral load of subjects was 148,141 copies/ml (interquartile range 1,294-1,157,417 copies/mL), and the median CD4+ T cell count was 230 cells/μl (interquartile range 46-349 cells/μl). The median CD8+ T cell count was 1053 cells/μl (interquartile range 382-1675). And most of the subjects were men who have sex with men (MSM).

### Changes of CD8+ T cells, naïve, CM, EM and EMRA subsets

**Figure 1:**
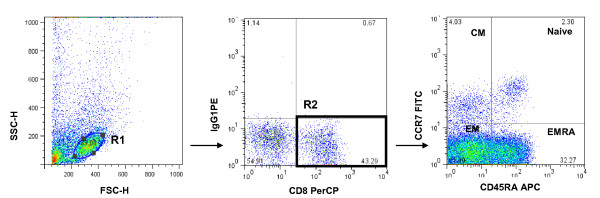
**The gating strategy of CD8+ T cell populations from a single representative subject**. Lymphocytes were gated first. Then CD8+ cells were gated. Central memory (CM; CD45RA-CCR7+), naive (CD45RA + CCR7+), effector memory (EM; CD45RA-CCR7-), and terminally differentiated effector (EMRA; CD45RA + CCR7-) subsets were gated based on gated CD8+ cells.

**Figure 2:**
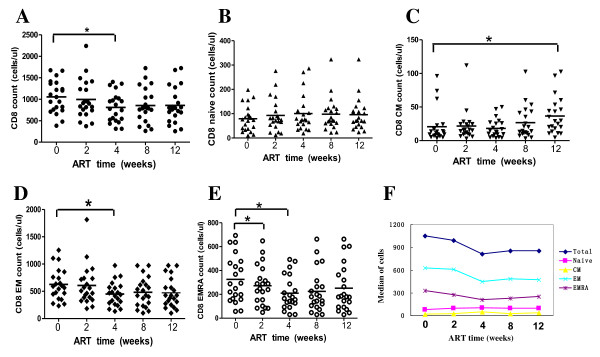
**Percentage changes in absolute CD8+ T cells and each phenotypic subset during initial ART (weeks)**. (A-E) Percentage changes of CD8+ T cells, CD8 CM, CD8 EM and CD8 EMRA subsets respectively. (F) Mean percentage changes of CD8+ T cells, CD8 CM, CD8 EM and CD8 EMRA subsets.

The gating strategy of CD8+ T cell populations was shown in figure 1. Longitudinal analyses of CD8+ T cells, naïve, CM, EM and EMRA subsets in patients with asymptomatic chronic HIV infection after ART were shown in figure 2. The major components of CD8+ cells were EM and EMRA subsets, which accounted for over 80 percent. Naïve and CM subsets only accounted for less than 20 percent.

The median of CD8+ T cells decreased from 1053 to 904 cells/μl after 12 weeks of ART. Among the four subsets of CD8+ cells, CD8+ EM and CD8+ EMRA subsets had the same change pattern with CD8+ T cells. The median of CD8+ EM subset decreased significantly from 627 to 520 cells/μl. Similarly, the median of CD8+ EMRA subset decreased significantly from 325 to 272 cells/μl.

The median of CD8+ naïve cells at baseline, week 2, 4, 8 and 12 was 79, 91, 99, 98, 102 cells/μl respectively. There were no significant differences among them. The median of CD8+ CM subset kept slowly rising, from 21 cells/μl at baseline to 43 cells/μl at week 12 after ART.

### Activation of CD8+ cell subsets

We next investigated the effect of ART on T cell activation. HIV-infected individuals had higher T-cell activation in the blood as indicated by expression of HLA-DR and CD38 [9]. Given the limited experimental conditions, we did not stain CD38 with HLA-DR simultaneously. At baseline the median proportion of activated CD8+ T lymphocytes (CD38+) exceeded 80% and gradually declined over 12 weeks, reaching 73.77% (73.77 ± 9.14) at the last follow up visit. Activation of CD8+ EM subsets decreased in a similar way, from 83.53% at baseline to 72.87% at week 12. The median of activation of CD8+ naïve cell subset was 62.997% at baseline. It fluctuated between 49.09% and 65.79%, reaching to 63.32% at week 12.

The median of percentage of CD38+ CD8+ CM subset was 57.81%, 55.63%, 52.82%, 54.49% and 50.18% respectively at the 5 times of follow up visits, fluctuating between 50% and 58%. The median of CD8+ EMRA subset was 84.43%, 86.11%, 83.64%, 85.22% and 83.90% respectively at the 5 times of follow up visits, staying at a high level. Activation levels of the two subsets had no significant changes after ART.

With respect to HLA-DR expression on CD8+ lymphocytes, there was also a high percentage of expression at base line. The percentage of HLA-DR expression decreased from 76.91% at baseline to 71.26 at week 12 after ART, which has the similar change pattern to that of CD38 expression.

The median of HLA-DR expression on CD8+ naïve cell subset declined from 9.39% at baseline to 7.24% at week 12 after ART. For CD8+ CM subset, the median percentage of HLA-DR expression declined from 65.91% at baseline to 51.43% at week 12. The median percentages of CD8+ EM and EMRA subsets were both in a high level around 80%. There were no significant changes in activation of CD8+ EM and EMRA subsets as indicated by HLA-DR.

### Proliferation of CD8+ cell subsets

Proliferating subsets are calculated by measurement of Ki67 expression. The median of percentage of Ki67+CD8+ T cells elevated slightly at the first 4 weeks of ART, then decreased gradually. Their values at the 5 times of follow up visits were 4.85%, 7.27%, 6.85%, 5.73% and 4.27% respectively.

The median of proliferation of CD8+ naïve subset was 1.29%, 1.47%, 1.33%, 0.83% and 0.85% respectively at the 5 times of follow up visits. And the median of proliferation of CD8+ CM subset was 7.47%, 8.05%, 8.52%, 5.41% and 4.40% respectively. Both of the two subsets had the trend of decline.

Ki67 expression on CD8+ EM cells had no significantly change after ART, fluctuating between 5.60% and 9.65%. For CD8+ EMRA cells, percentage of Ki67 positive cells elevated after ART, reaching to peak of 4.09% at week 8, then decreased to 2.74% at week 12.

The changes of mean fluorescence index (MFI) of CD38 and Ki67 on CD8+ cell subsets have the same pattern with those of percentages (data not shown).

## Discussion

It has been reported previously that changes in the levels of T cell subsets occurred after a long-term of ART, showing a biphasic increase in CD4+ T cells and a trend of decrease in CD8+ T cells [4-6]. However, little is known about the change of CD8+ cell subsets during early period of ART. In this study we investigated the dynamic changes not only in CD8+ cell subsets, but also in their activation and proliferation subsets in Chinese HIV/AIDS patients during early period of ART, particularly in MSM population.

The number of CD 8+ T cells decrease after long-term of ART [10,11]. Since their follow-up intervals are too long, the early dynamic of CD8+ T cells can not be presented completely. Here, our results displayed a complete phenotypical picture of CD8+ cell subsets during initial ART. The total CD8+ T cells had a tendency of decrease (see figure 2).

Of the 4 subsets we studied, EMRA and EM subsets declined in consistent with total CD8+ T cells, while the naïve and CM subsets had a tendency of increase during the first 3 months of ART (see figure 2). However, most of the changes had no significant differences. From the results we can see that the declines of CD8+ T cells are mainly composed of EMRA and EM subsets, which may play important roles in direct killing of target cells. The decrease of EMRA and EM subsets may result from the migration of these cells from blood to lymph tissues [12]. Another reason may be the decline of HIV antigens after ART [13,14].

During progressive HIV infection, naive T cells are preferentially targeted, causing a marked decrease in their proportion [15,16]. The process of immune recovery in HAART-treated adults induces a slow sustained increase of naive lymphocytes [13,17]. The memory subset derives from the naive cells by a post-thymic maturation process. Our results showed that the naïve and CM subsets have a tendency of increase. The increase of naïve and CM subsets may originate from non-HIV specific CD8 cells, as was previous reported [18] that CD8+ memory cells increased following HAART were not considered as HIV-specific T cells.

**Figure 3:**
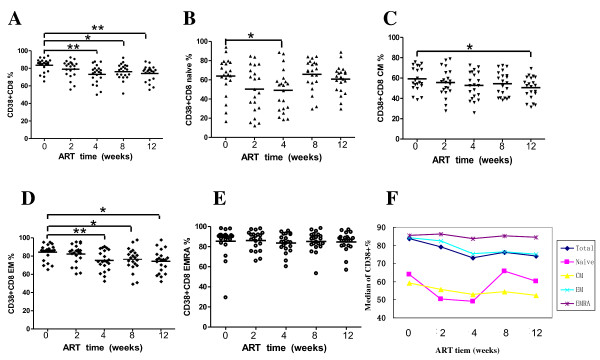
**Percentage changes in CD38 expressed CD8+ T cells and each phenotypic subset during initial ART (weeks)**. (A-E) Percentage changes of CD38 expressed CD8+ T cells, CD8 CM, CD8 EM and CD8 EMRA subsets respectively. (F) Mean percentage changes of CD38 expressed CD8+ T cells, CD8 CM, CD8 EM and CD8 EMRA subsets.

**Figure 4:**
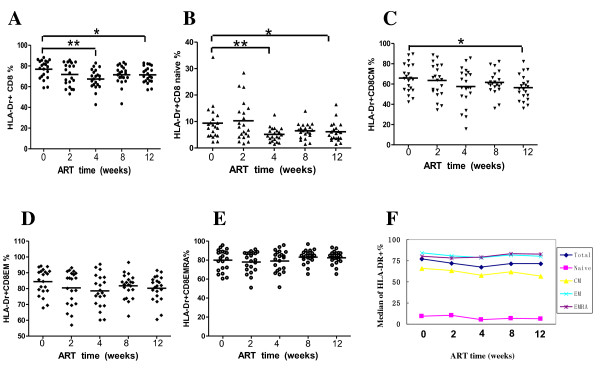
**Percentage changes in HLA-DR expressed CD8+ T cells and each phenotypic subset during initial ART (weeks)**. (A-E) Percentage changes of HLA-DR expressed CD8+ T cells, CD8 CM, CD8 EM and CD8 EMRA subsets respectively. (F) Mean percentage changes of HLA-DR expressed CD8+ T cells, CD8 CM, CD8 EM and CD8 EMRA subsets.

Activation of T cells is an important pathogenetic event in HIV infection, which can be indicated by the elevated expression of different antigens like CD38 and HLA-DR on the surface of T lymphocytes [9]. CD38 level is a strongest predictive marker of HIV disease progression [19,20] and may even predict antiretroviral therapy (ART) treatment failure [21,22]. Several studies [23-25] showed activated CD8+ T cells decreased after ART. But they did not reveal the change of activated CD8+ cell subsets. Both CD38 and HLA-DR expression on CD8+ cell subsets decreased after ART in our study. However, the magnitude of decrease is not remarkable in some subsets of CD8+ T cells, especially EMRA subset (see figure 3 and 4). One explanation is that the plasma HIV viral loads are still above lower detection limit (LDL) in most patients after 3 month of ART (data not shown). Another reason is the potential exist of various opportunistic viral and bacterial infections.

**Figure 5:**
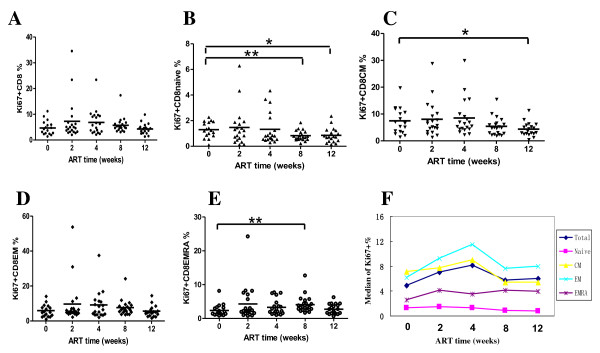
**The changes in Ki67 expressed CD8+ T cells and each phenotypic subset during initial ART (weeks)**. (A-E) changes of Ki67 expressed CD8+ T cells, CD8 CM, CD8 EM and CD8 EMRA subsets respectively. (F) Mean changes of Ki67 expressed CD8+ T cells, CD8 CM, CD8 EM and CD8 EMRA subsets.

T-cell proliferation based on Ki67 expression is correlated generally with those obtained using direct techniques [26,27], such as [2H] glucose incorporation. There are some differences on the changes of percentage of ki67+ CD8+ T cells. One report showed that the percentages of CD8+Ki67+ cells increased during ART [28], and the subset was maintained at a high percentage until 18 weeks post ART. Another report [29] demonstrated a decline of percentages of CD8+Ki67+ cells. Our results showed that the percentages of Ki67+CD8+ cells as well as EM and EMRA subsets increased, while those of naïve and CM subsets decreased (see figure 5). The differences may come from sources of patients, stage of the disease and duration of treatment time.

In conclusion, the changes of CD8+ T cell subsets during initial ART are complex. Almost all of the CD8+ cell subsets declined in activation levels during initial ART. However, the trends of proliferation levels in different CD8+ subsets were inconsistent. Further studies are needed to perform on a large scale and general population.

## Materials and methods

### Participants and study

Twenty-one HIV-1-infected treatment-naïve patients were randomly enrolled from HIV/AIDS clinic of Beijing You'an Hospital, with CD4+ T cell counts at less than 350 cells/ul and no opportunistic infections within the previous three months. Exclusion criteria included pregnancy, active tuberculosis, or serious liver/renal dysfunction. All individuals were treated with ART, which included 3TC + d4T (or AZT) + NVP. The study was approved by the Beijing You'an Hospital Research Ethics Committee, and written informed consent was obtained from each subject.

### Collection of Blood Samples

Fasting venous blood samples were collected at 8-9 in the morning in EDTA-containing tubes at baseline, 2, 4, 8 and 12 weeks of treatment. Peripheral blood mononuclear cells (PBMCs) were isolated by Ficoll-Hypaque density gradient centrifugation. Activation and proliferation markers were detected immediately after isolation.

### Flow cytometric analysis

The monoclonal antibodies (mAbs) of CD8+-PerCP, CCR7-FITC, HLA-DR-PE and CD38-PE were purchased from BD Bioscience (San Diego, CA, USA). Anti-CD45RA-APC and anti-Ki67-PE was purchased from eBioscience (San Diego, CA, USA).

Levels of immune activation were examined through CD38 and HLA-DR expressions on different subsets of CD8+ T lymphocytes. Freshly isolated PBMCs were stained with ant-CD8-PerCP, anti-CD45RA-APC, anti-CCR7-FITC and anti-CD38-PE or anti-HLA-DR-PE or the corresponding IgG1-PE isotype control according to manufacturer's instructions.

Cell proliferation was studied by measuring expression of the Ki-67 antigen. Freshly isolated PBMCs were incubated with anti-CD8-PerCP, anti-CD45RA-APC, anti-CCR7-FITC at 4°C for 30 min according to manufacture's instructions. After washing with phosphate-buffered saline (PBS), permeabilization was performed by incubating cells with Cytofix/Cytoperm (BD Pharmigen, San Diegio, CA) at 4°C for 20 min. Cells were stained intracellulary with anti-Ki67-PE or the corresponding isotype control anti-IgG1-PE at room temperature for 30 min. After washing with PBS, Four-color flow cytometric analyses were then performed using FACSCalibur and CELLQuest software (Becton Dickinson, San Jose, CA).

### Assays for CD4+ and CD8+ T cell counts and Plasma HIV-1 RNA

After whole-blood lysis (FACSlysing Solution, Becton Dickinson San Diego, CA, USA), T lymphocyte counts were determined by three-color flow cytometry using CD3-APC, CD4-FITC and CD8+-PE monoclonal antibody (BD Bioscience San Diego, CA, USA). The analysis was performed on a BD FACSCount flow cytometer in accordance with Chinese Center for Disease Control and Prevention (CDC) guidelines.

Plasma viral load were measured by the Amplicor HIV-1 monitor ultrasensitive Method (Roche, Germany), with a detection limit of 40 copies/ml of plasma.

### Statistical analysis

Data analysis was performed with SPSS 11.5 for Windows software (SPSS Inc, Chicago, IL). Statistical significance within groups was analyzed with Kruskal-Wallis Test; statistical significance between groups was analyzed with the Mann-Whitney U test. P < 0.05 is considered statistically significant.

## Competing interests

The authors declare that they have no competing interests.

## Authors' contributions

YJ drafted the manuscript and statistical analyses. WH participated in flow cytometric analysis. TZ followed up patients and collected samples. YZ assisted with manuscript and data anlysis. YJ assisted with flow cytometric analysis and data acquisition. HZ conceived the study and participated in the data analysis. HW supervised and coordinated the study. All authors have read and approved the final manuscript.
